# Effects of daily ingestion of sodium bicarbonate on acid-base status and anaerobic performance during an altitude sojourn at high altitude: a randomized controlled trial

**DOI:** 10.1186/s12970-020-00351-y

**Published:** 2020-04-19

**Authors:** Mirjam Limmer, Markus de Marées, Petra Platen

**Affiliations:** 1 Department of Sports Medicine and Sports Nutrition, Ruhr-Universität Bochum, Gesundheitscampus Nord 10, 44801 Bochum, Germany; 2 Institute of Outdoor Sports and Environmental Science, German Sport University Cologne, Cologne, Germany

**Keywords:** Chronic sodium bicarbonate supplementation, Baking soda, Moderate altitude, High-intensity exercise, Portable tethered sprint running test, Hypobaric hypoxia

## Abstract

**Background:**

The present study investigated the effects of chronic sodium bicarbonate (NaHCO_3_) ingestion on a single bout of high-intensity exercise and on acid-base balance during 7-day high-altitude exposure.

**Methods:**

Ten recreationally active subjects participated in a pre-test at sea level and a 7-day hiking tour in the Swiss Alps up to 4554 m above sea level. Subjects received either a daily dose of 0.3 g/kg NaHCO_3_ solution (*n* = 5) or water as a placebo (*n* = 5) for 7 days. Anaerobic high-intensity exercise performance was assessed using the portable tethered sprint running (PTSR) test under normoxic and hypoxic conditions (3585 m). PTSR tests assessed overall peak force, mean force, and fatigue index. Blood lactate levels and blood gas parameters were assessed pre- and post-PTSR. Urinary pH and blood gas parameters were further analyzed daily at rest in early morning samples under normoxic and hypoxic conditions.

**Results:**

There were no significant differences between the bicarbonate and control group in any of the PTSR-related parameters. However, urinary pH (*p* = 0.003, ηp^2^ = 0.458), early morning blood bicarbonate concentration (*p* < 0.001, ηp^2^ = 0.457) and base excess (*p* = 0.002, ηp^2^ = 0.436) were significantly higher in the bicarbonate group compared with the control group under hypoxic conditions.

**Conclusions:**

These results indicate that oral NaHCO_3_ ingestion does not ameliorate the hypoxia-induced impairment in anaerobic, high-intensity exercise performance, represented by PTSR-related test parameters, under hypobaric, hypoxic conditions, but the maximal performance measurements may have been negatively affected by other factors, such as poor implementation of PTSR test instructions, pre-acclimatization, the time course of hypoxia-induced renal [HCO_3_^−^] compensation, changes in the concentrations of intra- and extracellular ions others than [H^+^] and [HCO_3_^−^], or gastrointestinal disturbances caused by NaHCO_3_ ingestion. However, chronic NaHCO_3_ ingestion improves blood bicarbonate concentration and base excess at altitude, which partially represent the blood buffering capacity.

## Background

Consistent physical performance is indispensable for athletes during altitude training and for mountaineers climbing at moderate and high altitudes. Several studies have shown that altitude training can improve athletic performance, and it has thus become an accepted training method in various types of sports [1–3]. Furthermore, increasing numbers of people are travelling to moderate or high altitudes for trekking, climbing, mountaineering, or skiing activities. However, acute exposure to moderate and high altitudes above 1500 m acutely impairs physical performance [4]. Reduced exercise tolerance at altitude is caused by severe disruption to homeostasis resulting from a decline in arterial oxygen saturation (s_a_O_2_) due to reduced oxygen pressure in the ambient and inspired air (P_I_O_2_) [5]. The reduced P_I_O_2_ leads to an increased respiratory rate, resulting among others in an acute respiratory alkalosis. Subsequent renal compensation of the acute respiratory alkalosis induces a reduction in blood bicarbonate ([HCO_3_^−^]) concentrations, and the resulting decline in the blood buffering capacity during altitude adaption has been suggested to have a significant effect on exercise performance at altitude, particularly above the lactate threshold [6–9]. These findings are supported by the fact that exercise-induced acidosis is more severe at altitude compared with sea level [6, 10].

Dietary strategies have previously been used to attenuate the impaired exercise performance under hypoxic conditions [11–13]. Among these, supplementation with sodium bicarbonate (NaHCO_3_) as an alkalotic buffer is an interesting approach to alleviate the elevated acidic stress during exercise above the lactate threshold under hypoxic conditions [11]. In normoxic conditions, NaHCO_3_ is proposed to enhance anaerobic exercise performance by increasing the availability of blood [HCO_3_^−^], thereby strengthening the physiochemical buffering capacity to reduce the rate of hydrogen cation ([H^+^]) production during exercise [11, 14, 15]. However, while the results of some studies have suggested that significant increases in [H^+^] may impair subsequent exercise performance by reducing the capacity for muscle force production [16, 17], the results of other studies suggest that exercise performance may be unaffected by disturbances in acid-base balance [18]; thus, there remains no consensus in the literature regarding the roles of acid-base status in anaerobic exercise performance and skeletal muscle fatigue. In addition, it has been suggested that the relative increase in glycolytic flux under hypoxic conditions increases [H^+^] production, which may be the underlying mechanism for the beneficial effect of NaHCO_3_ ingestion under hypoxic conditions [16, 19]. Furthermore, the removal of [H^+^] may be inhibited and the blood [HCO_3_^−^]-buffering capacity may be reduced [16, 19], due to the proposed lower [HCO_3_^−^] concentrations present under hypoxic conditions [8].

Few studies have examined the effect of NaHCO_3_ ingestion on anaerobic exercise performance at altitude, and the results of recent studies have been inconsistent. Flinn et al. [20] and Saunders et al. [21] found no effect of NaHCO_3_ supplementation on the power output of intermittent high-intensity exercise at simulated altitudes of 3000 m and 2500 m, respectively, while several studies have supported the assumption of a beneficial effect of NaHCO_3_ ingestion on anaerobic performance at altitude. Feriche Fernandez-Castanys et al. [22], Hauswirth et al. [23], and McLellan et al. [24] all described increased or constant exercise performance under acute altitude conditions in hypoxic chambers compared with sea-level performance in subjects receiving alkalizing agent supplements prior to exercise. In addition, Deb et al. [11, 19] and Gough et al. [16] reported positive effects of NaHCO_3_ under acute moderate hypoxic conditions at simulated altitude during intermittent and repeated high-intensity exercise. They conclude that NaHCO_3_ ingestion may offer an ergogenic strategy to mitigate hypoxia-induced declines in exercise performance. Most previous studies tested the effect of acute ingestion of a single dose of NaHCO3 shortly before exercise testing on anaerobic exercise performance, but the effects of chronic daily ingestion of NaHCO3 on anaerobic performance have also been evaluated. However, although chronic ingestion of NaHCO3 has been suggested to improve high-intensity work more effectively than acute ingestion [25–28] at sea level, especially during several-day training schedules, to the best of our knowledge, the influence of chronic NaHCO3 ingestion on exercise performance under hypoxic conditions has not been investigated to date. The aforementioned findings on the effects of NaHCO_3_ ingestion prior to high-intensity exercise under simulated altitude conditions raise questions regarding the potential practical implications of these results for mountaineering disciplines with a high anaerobic demand performed under hypobaric hypoxic conditions over a period of several days. Although mountaineering is mainly associated with aerobic performance [29], several disciplines are performed at moderate to high altitudes with high anaerobic demands, such as cross-country skiing [30, 31], alpine ski racing [32, 33], cross-country mountain biking and transalpine challenges [34, 35], and single- und multi-pitch climbing [36, 37]. These activities are affected by reduced exercise performance at altitude due to hypoxia, and may thus benefit from NaHCO_3_ ingestion to improve anaerobic exercise performance. However, there have been few studies regarding the beneficial effects of NaHCO3 ingestion during several-day altitude sojourns with high applicability to sport activities performed at moderate to high altitudes.

The present study aimed to analyze the effects of chronic ingestion of NaHCO_3_ on a single bout of high-intensity exercise and on the acid-base status during high-altitude exposure for 7 days. We hypothesized that chronic NaHCO_3_ ingestion would attenuate the hypoxia-induced impairment in anaerobic, high-intensity exercise performance under hypobaric, hypoxic conditions. We further hypothesized that daily chronic NaHCO_3_ ingestion would increase urinary pH levels and blood gas parameters during an altitude sojourn.

## Methods

### Participants

Fourteen healthy, non-specifically trained adult volunteers participated in the present study. Of these, four dropped out during the study due to medical problems (two because of moderate acute mountain sickness and two because of orthopedic problems). The results for the remaining 10 participants were analyzed (bicarbonate group: 4 men and 1 woman; control group: 4 men and 1 woman). Anthropometric data for participants in the bicarbonate and control groups are shown in Table 1. All participants underwent a medical screening before entering the study. Participants had to be in good health with no pre-existing altitude illnesses, cardiac or pulmonary conditions, and no musculoskeletal injuries that could interfere with mountaineering or running activities. All participants lived close to sea level. Because of the assumption that NaHCO_3_ [38] has a greater effect in recreationally trained compared with specifically trained persons, we set moderate physical fitness as an inclusion criterion. Exclusion criteria included acute muscular injuries or restrictions, chronic medication intake, alcohol consumption, acute infections, and preceding altitude sojourns above 2000 m in the 4 weeks prior to the investigation. The study was approved by the ethical committee of the Ruhr-Universität Bochum in accordance with the Declaration of Helsinki. Subjects gave their written informed consent after they had been informed of all experimental procedures and risks. Subjects were randomly assigned to either the bicarbonate supplementation (BIC) or the control group (CON).

**Table 1:** Anthropometric data for subjects in the bicarbonate supplementation and control groups

	BIC (male: ***n*** = 4; female: ***n*** = 1)	CON (male: ***n*** = 4; female: ***n*** = 1)
*Age (years)*	25.0 ± 3.2	23.2 ± 2.3
*Body mass (kg)*	69.0 ± 12.2	73.3 ± 9.5
*Height (cm)*	173.7 ± 10.0	179.1 ± 7.9
*BMI (kg/m*^*2*^*)*	22.8 ± 1.7	22.8 ± 1.0

Data presented as mean ± standard deviation. *BIC* Bicarbonate supplementation, *CON* Control, *BMI* Body mass index. For further details see Materials and Methods section

### Experimental design

**Fig. 1:**
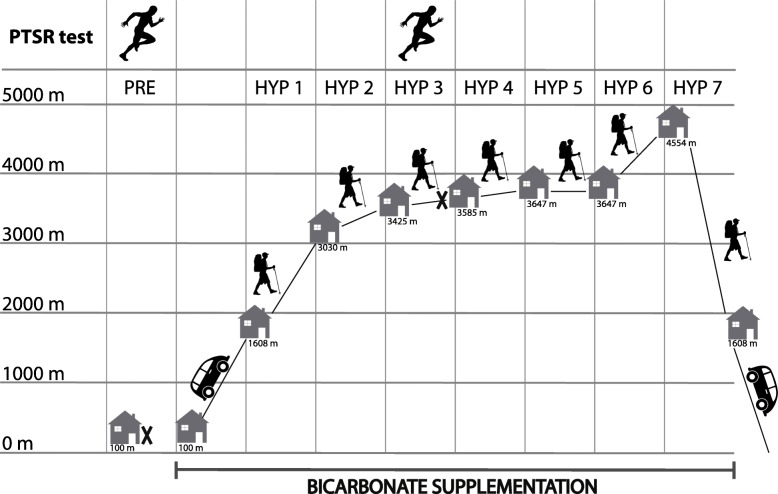
Experimental sequence. Sleeping heights and days of PTSR tests during a 7-day mountaineering tour. NOR = nomoxia, HYP = hypoxia, PTSR = portable tethered sprint running test

All test persons participated in a 7-day mountaineering tour (HYP1 – HYP7) at moderate to high altitude in the European Alps (Wallis, Switzerland). Glacier trekking was accomplished up to 4554 m above sea level and the sleeping heights were between 3030 m and 4554 m above sea level. Each participant passed a baseline test at sea level under normoxic conditions (NOR) before altitude exposure (elevation = 100 m). Anaerobic performance tests were performed at sea level (NOR) and after 3 days of altitude exposure (HYP3) at 3585 m above sea level (Fig. 1).

### Supplementation

Based on commonly-used NaHCO_3_ doses [15, 39–42], the bicarbonate group received a NaHCO_3_ dose of 0.3 g/kg body mass daily, dissolved in 1 l water. On exercise testing days, NaHCO_3_ was administered 1 h before exercise testing. The control group drank 1 l water in the same time, as a placebo treatment. The groups received the respective treatments for 8 days, starting on the day when travelling to altitude exposure. After our subjects reported GI discomfort and GI symptoms seem to increase with increasing NaHCO_3_ dose, we decided to reduce the dose to 0.15 g/kg body mass on day five of supplementation.

### Anaerobic performance test

Anaerobic performance was measured using a portable test device during the tethered sprint running (PTSR) test [43]. The PTSR test was chosen because it is simple, requires little space, and does not involve heavy and unwieldy equipment. These aspects were important because of the need to carry the test equipment and carry out the anaerobic testing under hypoxic conditions in the restricted spatial conditions of a mountain hut. Furthermore, NaHCO3 ingestion is recommended to enhance anaerobic exercise performance for short durations of about 1 min [41] and work performed at high intensity during continuous exercise has been shown to be enhanced by prior NaHCO_3_ ingestion [44]. We therefore assessed anaerobic performance using a single PTSR test of 60-s duration. For the test, participants ran with a belt round the waist to collect force measurements. The belt was attached to an inextensible static rope combined in series with a load cell and fixed to a pillar at a 90° angle to the subject’s waist height. Following a structured 10-min warm-up, the participant performed an all-out sprint for 60 s. Participants were instructed to sprint at maximal effort and pull the rope with full force. Study investigators provided strong verbal encouragement for the entire test duration and fluids were provided ad libitum, both before and after the 60-s sprint period. Force data were recorded in Newton (N) and the variables overall peak force (PF), lowest force (F_min_), and mean force (MF) were determined over the 60-s test duration. The PF was taken to be the highest 5-s force and F_min_ the lowest 5-s force during the 60-s sprint period. MF was determined as the mean value over the entire 60-s test, and PF and MF were used in subsequent analyses. The fatigue level during the PTSR test was assessed using the fatigue index (FI), which was calculated as FI (%) = [(PF – F_min_)/PF] × 100, as recommended for Wingate tests [43]. Blood lactate levels were measured in 20-μl capillary blood samples collected before and 2, 4, 6, 8 and 10 min after PTSR testing. The samples were cooled and stored for up to 7 days until analysis (BIOSEN S-Line, EKF Diagnostics, UK). The maximum post-exercise lactate concentration (La_max_) of one PTSR test was used for statistical analyses.

### Blood gas analysis

Capillary blood samples (100 μl) were taken from a hyperemized earlobe daily before breakfast (HYP1 – HYP7) and pre- and post-PTSR tests. Blood samples were immediately analyzed for blood gas parameters using a portable epoc® blood gas analyzer (Alere GmbH, Cologne, Germany). The oxygen and carbon dioxide partial pressure (*P*O_2_/*P*CO_2_), blood pH (pH_b_), oxygen saturation (s_a_O_2_), blood bicarbonate concentration ([HCO_3_^−^]) and base excess (BE) were determined. We further calculated the difference between pre- and post-PTSR values (DIFF PTSR) for all blood gas parameters.

### Urine pH

Urinary pH (pH_u_) was determined each day in spontaneous early morning urine samples (at least 5 ml of urine) using Neutralit® pH-indicator strips pH 5.0–10.0 (Merck, Darmstadt, Germany). pH_u_ was measured throughout the experimental period (NOR; HYP1-HYP7) and served as a surrogate marker to assure that the supplementation had been conducted successfully [45].

### Lake Louise AMS score

Acute mountain sickness (AMS) was assessed each morning using the Lake Louise AMS score (LLS) to ensure that it was assessed only after the minimum recommended altitude exposure of at least 6 h [46]. The LLS is a self-report questionnaire assessing the symptoms of headache, nausea/vomiting, fatigue, and dizziness/light-headedness. Each symptom item is scored from 0 (not present) to 3 (severe), and the sum score for the four symptom items was calculated (0–12 points) and used for statistical analyses. An individual sum score ≥ 3 and a headache score of ≥1 was mandatory for a positive AMS score. Mild AMS was defined as a LLS of 3–5 points, moderate AMS as 6–9 points, and severe AMS as 10–12 points, with headache and a recent altitude gain as conditions [46].

### Body weight

Body weight was measured daily before breakfast using digital scales (Seca Clara 830, Seca Germany, Hamburg, Germany).

### Statistical analysis

Data are presented as mean ± standard deviation. All departures from a normal distribution were identified using the Shapiro-Wilk test. We calculated ∆ values (HYP3 minus NOR) for all PTSR-related parameters to identify the influence of hypoxia on the respective parameters. Differences in ∆ values between BIC and CON conditions were assessed by two-sample *t*-tests. Cohen’s d (*d*) was used to calculate effect sizes, with 0.2 considered to indicate a small effect, 0.5 a medium effect, and 0.8 a large effect [47]. Non-normally distributed variables (∆ *P*CO_2_ PRE PTSR) were analyzed using Mann-Whitney-U-tests and effect sizes were calculated using correlation coefficients (r). The effect of conditions (BIC and CON) on the parameters *P*O_2_, *P*CO_2_, s_a_O_2_, pH_b_, [HCO_3_^−^], BE, pH_u_, LLS, and body weight over time (NOR vs. HYP1 – HYP7) and pre and post-PTSR (NOR PRE PTSR vs. NOR POST PTSR; HYP PRE PTSR vs. HYP POST PTSR) were tested by two-way [condition x time] repeated-measures ANOVA. Violations of the assumption of sphericity were corrected for by Greenhouse-Geisser adjustments. Two-tailed *t*-tests were utilized as post hoc tests to indicate significant differences. A Bonferroni procedure was used (p*) to retain α = 0.05, and the significance level was set at *p* ≤ 0.05 in all comparisons. Effect sizes were calculated using partial η squared (ηp^2^) and interpreted as small (0.01), medium (0.06), and large (0.14), respectively [48]. The α level was set at *p* ≤ 0.05, and all analyses were conducted using SPSS 25 (IBM Corp., Armonk, NY, USA). The free software G*Power [49] was used to calculate required sample sizes and effect sizes.

## Results

### Anaerobic performance test

**Fig. 2:**
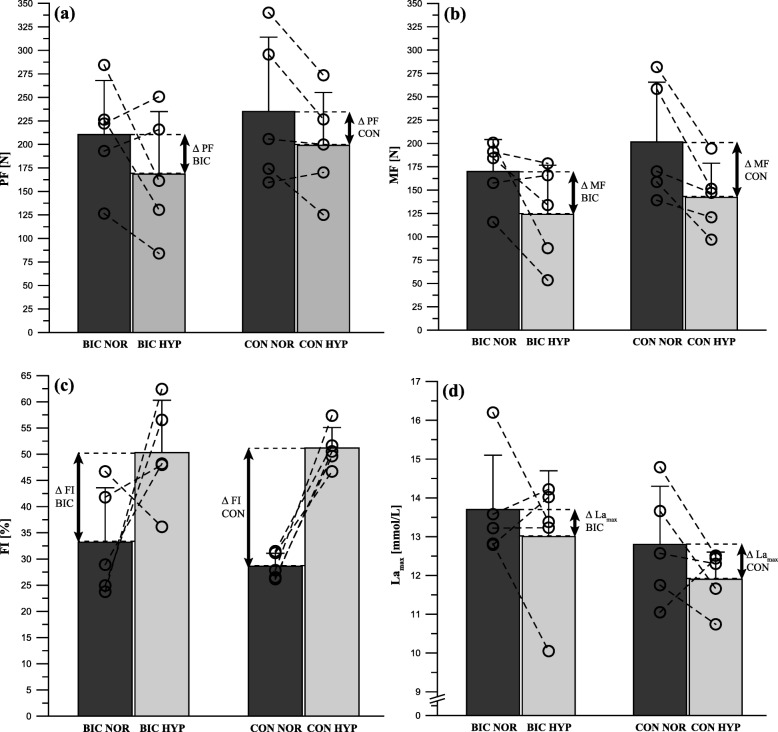
Performance measurements with (BIC) and without (CON) bicarbonate supplementation under normoxic (NOR) and hypoxic (HYP) conditions for (**a**) peak force (PF), (**b**) mean force (MF), and (**c**) fatigue index (FI), as well as the associated physiological response (**d**) maximum blood lactate (La_max_). Statistical analyses are for ∆ values only (HYP minus NOR), indicating intra-individual hypoxic-induced changes in performance parameters. Data points represent individual values (○). Bar charts are mean ± standard deviation. See Materials and methods for further details

There were no significant differences between the bicarbonate and control groups in ∆PF (BIC: − 42.0 ± 68.3, CON: − 36.0 ± 36.3 N; *p* = 0.866, *d* = 0.11), ∆MF (BIC: − 46.0 ± 47.0, CON: − 59.5 ± 38.9 N; *p* = 0.634, *d* = 0.31), and ∆FI (BIC: 17.0 ± 19.8, CON: 22.5 ± 5.2%; *p* = 0.575, *d* = 0.38) (Fig. 2 a−c). There was also no difference between the groups in ∆La_max_ (BIC: − 0.7 ± 1.9, CON: − 0.8 ± 1.5 mmol/L; *p* = 0.935, *d* = 0.05) (Fig. 2 d).

### Blood gas analysis

In term of blood gas parameters, there were significant differences between the two groups for ∆[HCO_3_^−^] PRE PTSR (*p* = 0.004, *d* = 2.53), ∆BE PRE PTSR (*p* = 0.001, *d* = 3.15), ∆pH_b_ POST PTSR (*p* = 0.003, *d* = 2.65), ∆[HCO_3_^−^] POST PTSR (*p* = 0.001, *d* = 3.14), and ∆BE POST PTSR (*p* = 0.001, *d* = 3.16). There was also a significant difference for ∆pH_b_ DIFF PTSR (*p* = 0.032, *d* = 0.30) (Table 2).

**Table 2:** PTSR-related blood gas parameters in the bicarbonate supplementation and control groups under normoxia and hypoxia, and delta values

			***P***O_**2**_ [mmHg]	***P***CO_**2**_ [mmHg]	s_**a**_O_**2**_ [%]	pH_**b**_	[HCO_**3**_^**−**^] [mmol/L]	BE [mmol/L]
**N** **O** **R**	**BIC**	PRE PTSR	99.6 ± 23.2	32.2 ± 10.7	97.6 ± 1.2	7.44 ± 0.04	21.7 ± 6.1	−2.4 ± 5.6
		POST PTSR	94.4 ± 11.4	38.3 ± 4.8	95.8 ± 1.5	7.26 ± 0.02	17.4 ± 1.8	−9.7 ± 1.9
		DIFF PTSR	−5.2 ± 30.1	6.1 ± 11.0	− 1.8 ± 2.2	− 0.18 ± 0.04	− 4.3 ± 6.0	−7.3 ± 5.6
	**CON**	PRE PTSR	82.6 ± 7.9	38.7 ± 3.1	96.1 ± 1.4	7.42 ± 0.02	24.9 ± 1.3	0.3 ± 1.1
		POST PTSR	90.2 ± 7.9	38.7 ± 3.8	95.5 ± 1.1	7.26 ± 0.02	17.6 ± 2.0	− 9.5 ± 2.1
		DIFF PSTR	7.6 ± 9.7	− 0.0 ± 2.9	− 0.7 ± 1.6	− 0.15 ± 0.04	−7.3 ± 1.3	− 9.8 ± 1.7
**H** **Y** **P**	**BIC**	PRE PTSR	49.4 ± 2.5	31.6 ± 1.9	88.9 ± 1.7	7.52 ± 0.03*	26.0 ± 2.1*	3.2 ± 2.4*
		POST PTSR	57.3 ± 5.4	30.6 ± 1.8	89.4 ± 2.8	7.40 ± 0.03*	18.8 ± 1.2*	− 6.0 ± 1.6*
		DIFF PTSR	7.8 ± 4.9	− 1.0 ± 0.5	0.5 ± 2.6	− 0.13 ± 0.01*	−7.2 ± 0.9	− 9.2 ± 0.9
	**CON**	PRE PTSR	54.2 ± 5.1	28.4 ± 2.2	89.6 ± 3.0	7.46 ± 0.01	20.1 ± 1.3	− 3.8 ± 1.2
		POST PTSR	58.3 ± 3.3	28.4 ± 4.3	87.1 ± 1.6	7.29 ± 0.03	12.6 ± 1.5	− 14.1 ± 1.9
		DIFF PTSR	4.0 ± 7.7	0.0 ± 3.2	−2.6 ± 3.8	− 0.17 ± 0.03	−7.4 ± 1.6	−10.3 ± 1.9
∆	**BIC**	PRE PTSR	−50.2 ± 23.0	−0.6 ± 10.8	−8.7 ± 2.3	0.08 ± 0.06	4.4 ± 5.1*	5.6 ± 4.3*
		POST PTSR	−37.2 ± 13.0	− 7.7 ± 3.9	−6.5 ± 2.9	***0.14 ± 0.04***	1.5 ± 1.6*	3.7 ± 2.2*
		DIFF PTSR	13.0 ± 27.0	−7.0 ± 10.9	2.3 ± 1.6	0.06 ± 0.38*	− 2.9 ± 5.7	−1.9 ± 5.4
	**CON**	PRE PTSR	−28.3 ± 10.8	− 10.4 ± 1.8	−6.5 ± 3.6	0.04 ± 0.02	−4.8 ± 0.6	−4.1 ± 0.7
		POST PTSR	−31.9 ± 9.8	− 10.3 ± 4.1	−8.4 ± 2.2	0.02 ± 0.05	− 4.9 ± 2.4	−4.6 ± 3.0
		DIFF PTSR	−3.6 ± 16.9	0.1 ± 4.0	−1.9 ± 4.8	−0.02 ± 0.05	−0.1 ± 2.3	−0.5 ± 3.1

Data presented as mean ± standard deviation. *P*O_2_ Oxygen partial pressure, *P*CO_2_ Carbon dioxide partial pressure, *s*_*a*_*O*^*2*^ Oxygen saturation, *pH*_*b*_ Blood pH value, [*HCO*_*3*_^−^] Blood bicarbonate concentration, *BE* Base excess, *BIC* Bicarbonate supplementation group (*n* = 5), *CON* Control (*n* = 5), *NOR* Normoxia, *HYP* Hypoxia, *PTSR* Portable tethered sprint running test, *PRE PTSR* Pre-PTSR values, *POST PTSR* Post-PTSR values, *DIFF PTSR* Difference between pre- and post-PTSR values (DIFF = POST – PRE). ∆ = HYP minus NOR. For further details see Materials and Methods section. *p < 0.05 vs. CON. For *p*-values see results section

**Fig. 3:**
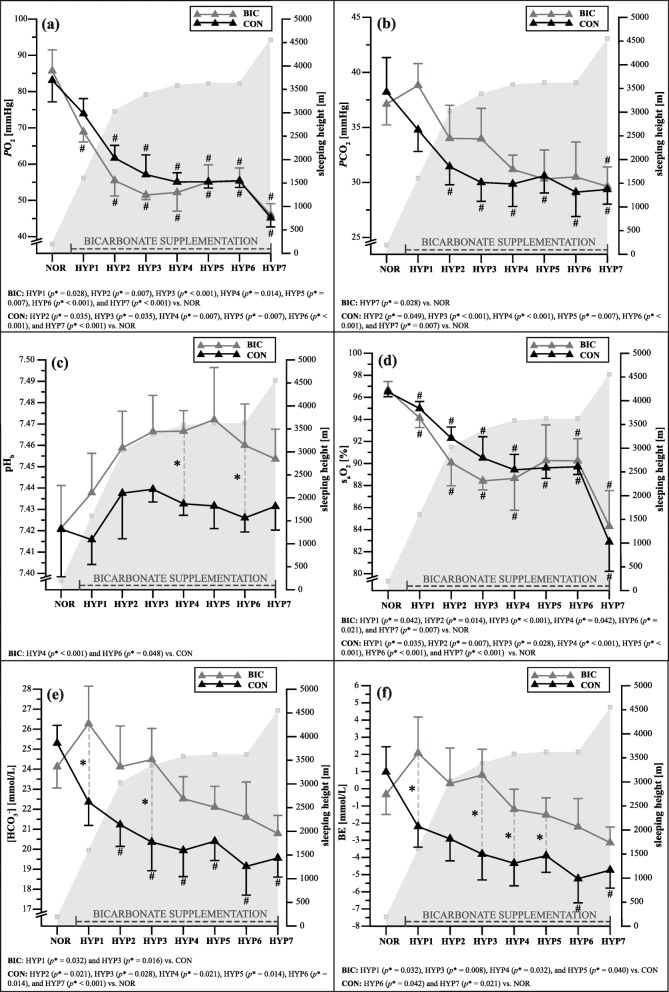
Early morning blood gas analysis measurements with (BIC) and without (CON) bicarbonate supplementation under normoxic (NOR) and hypoxic (HYP1-HYP7) conditions for (**a**) *P*O_2_, (**b**) *P*CO_2_, (**c**) pH_b_, (**d**) s_a_O_2_, (**e**) [HCO_3_^−^], and (**f**) BE. Data is presented as mean ± standard deviation. Filled gray graphs represent sleeping heights above sea level before the daily measurement. * *p* ≤ 0.05 vs. CON; # *p* ≤ 0.05 vs. PRE. See Materials and Methods for further details

The early morning blood gas analysis measurements for BIC and CON are shown in Fig. 3. A significant [condition x time] interaction was detected on *P*CO_2_ (*p* = 0.003, ηp^2^ = 0.306)_,_ [HCO_3_^−^] (*p* < 0.001, ηp^2^ = 0.457), and BE (*p* = 0.002, ηp^2^ = 0.436), but not for *P*O_2_*(p* = 0.176, ηp^2^ = 0.184), s_a_O_2_ (*p* = 0.227, ηp^2^ = 0.159), and pH_b_ (*p* = 0.263, ηp^2^ = 0.141). Additionally, the [condition] effect was significant for *P*O_2_*(p* = 0.047, ηp^2^ = 0.407), pH_b_ (*p* < 0.001, ηp^2^ = 0.912), [HCO_3_^−^] (*p* = 0.005, ηp^2^ = 0.646), and BE (*p* = 0.001, ηp^2^ = 0.743). A significant [time] effect was evident on change in *P*O_2_ (*p* < 0.001, ηp^2^ = 0.925), *PC*O_2_ (*p* < 0.001, ηp^2^ = 0.831), pH_b_ (*p* < 0.001, ηp^2^ = 0.399), s_a_O_2_ (*p* < 0.001, ηp^2^ = 0.867), [HCO_3_^−^] (*p* < 0.001, ηp^2^ = 0.774), and BE (*p* < 0.001, ηp^2^ = 0.695). Significant differences in pairwise comparisons and the associated *p*-values are shown in Fig. 3.

### Urine pH

**Fig. 4:**
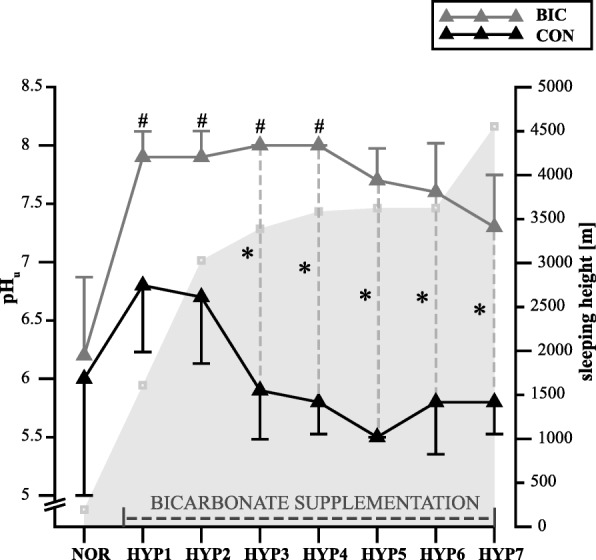
Early morning urinary pH values (means ± standard deviation) in subjects with (BIC) and without (CON) bicarbonate supplementation before (NOR) and during hypoxic exposure (HYP1-HYP7). * *p* ≤ 0.05 vs. CON; # *p* ≤ 0.05 vs. PRE. See Materials and Methods for further details

We found a main effect for pH_u_ (*p* = 0.003, ηp^2^ = 0.458) as well as a significant [condition] (*p* < 0.001, ηp^2^ = 0.945) and [time] effect (*p* = 0.001, ηp^2^ = 0.544). Post hoc analyses showed significant differences in pH_u_ between the bicarbonate and control groups at HYP3 (p* < 0.001), HYP4 (p* < 0.001), HYP5 (p* < 0.001), HYP6 (p* < 0.001), and HYP7 (p* < 0.001). Furthermore, there were significant differences in pH_u_ between NOR and HYP1 (p* = 0.049), HYP2 (p* = 0.049), HYP3 (p* = 0.028), and HYP4 (p* = 0.028) in the bicarbonate group, but not in the control group (Fig. 4).

### LLS

There was no main effect of interaction or effect of [condition] for LLS (BIC: HYP1: 2.0 ± 2.4, HYP2: 3.2 ± 2.4, HYP3: 2.4 ± 2.1, HYP4: 2.6 ± 3.0, HYP5: 2.0 ± 1.4, HYP6: 1.4 ± 1.5, HYP7: 1.8 ± 2.2; CON: HYP1: 1.0 ± 0.0, HYP2: 1.0 ± 0.7, HYP3: 0.8 ± 0.5, HYP4: 0.4 ± 06, HYP5: 0.4 ± 0.6, HYP6: 04 ± 0.6, HYP7: 0.6 ± 0.6; *p* = 0.266, ηp^2^ = 0.142), but there was a significant [time] effect for the LLS (*p* = 0.016, ηp^2^ = 0.268). Pairwise comparisons revealed a significant difference in LLS between HYP2 (2.1 ± 2.0) and HYP6 (0.9 ± 1.2, *p* = 0.049) for the total group.

### Body weight

There was no significant interaction [condition x time], or effect of [condition] or [time] for body weight during the experimental period in the bicarbonate group (NOR: 69.0 ± 12.2; HYP1: 70.1 ± 12.2; HYP2: 69.5 ± 11.8; HYP3: 70.1 ± 11.9; HYP4: 70.0 ± 12.3; HYP5: 70.1 ± 12.3; HYP6: 69.2 ± 12.0; HYP7: 69.4 ± 11.9 kg) and in the control group (NOR: 73.3 ± 9.5; HYP1: 73.4 ± 9.0; HYP2: 72.8 ± 9.9; HYP3: 72.5 ± 9.7; HYP4: 73.0 ± 8.8; HYP5: 72.8 ± 9.0; HYP6: 72.7 ± 8.5; HYP7: 72.5 ± 9.1 kg; *p* = 0.344, ηp^2^ = 0.127).

## Discussion

In the present study, we evaluated the effects of chronic NaHCO_3_ ingestion during 7 days of exposure to moderate and high altitudes on anaerobic performance parameters and laboratory blood and urine parameters. The primary finding of the study was that chronic NaHCO_3_ ingestion had a considerable impact on acid-base balance, which resulted in a higher alkalotic state; however, chronic NaHCO_3_ ingestion did not significantly influence PTSR-related performance outputs and associated physiological responses. A higher alkalotic acid-base balance prior to exercise under hypoxic conditions has been reported to be related to higher performance output and higher maximum blood lactate values after high-intensity exercise [11, 16, 19]. The suggested mechanism underlying the increased [H^+^] buffering from intramuscular to extramuscular compartments may lead to improved protection of intramuscular pH and increased anaerobic energy provision and glycogen utilization [16, 50]. However, although some outliers showed the expected increases in anaerobic performance outputs and the associated lactate response, the current study revealed no significant overall effect of chronic NaHCO_3_ ingestion on anaerobic performance outputs (indicated by ∆MF, ∆PF, and ∆FI) or on the related parameter ∆La_max_. This apparent discrepancy may be attributable to several factors.

Several recent studies reported increased or constant anaerobic exercise performance during acute altitude exposure in hypoxic chambers following supplementation with alkalizing agents prior to exercise [11, 16, 19, 22–24]. All these studies analyzed the effects of bicarbonate supplementation on different aspects of exercise performance under acute hypoxic conditions (between 15 min and 4 h of altitude exposure). In contrast, the present study assessed long-term altitude exposure over 7 days. We suggest that experimental acute hypoxia may not reflect the physiological acid-base responses to hypoxic conditions sufficiently, because the proposed lower [HCO_3_^−^] concentrations under hypoxic conditions [8] and the subsequently reduced [HCO_3_^−^] buffering capacity and anaerobic exercise performance [16, 17] should also take into account the time course of renal compensation of hypoxia-induced respiratory alkalosis, which is generally considered to be a slow-adapting mechanism affecting [HCO_3_^−^] concentrations after several hours or days. More specifically, renal [HCO_3_^−^] compensation has been shown to occur after 6 h and to be complete after 24 h of exposure to low to moderate altitudes, but to still be incomplete after 24 h of exposure to high altitudes [51]. In the present study, the enhanced renal [HCO_3_^−^] compensation was most obvious in the first 3 days of hypoxic exposure based on early morning urinary pH values. This finding complements the results of Ge et al. [51], who reported the renal [HCO_3_^−^] compensation based on early morning urinary pH values at simulated moderate altitudes of 1780, 2085, 2455 and 2800 m. They demonstrated that renal compensation was completed by 24 h at 1780, 2085 and 2455 m, but not at 2800 m. The time course of urinary pH values in our control group suggested that the renal [HCO_3_^−^] compensation at higher altitudes (between 2500 and 3500 m) seemed to be completed by 48 h. However, these results should be interpreted with caution because there was no significant increase of early morning urinary pH in the control group. This may be because of the high variability in pre-test urine and blood pH values. Urine and blood pH are influenced by several external factors such as nutrition, intake of dietary supplements, and high-intensity exercise [52]. Although subjects in the present study were asked to cease any special diets, supplements, and high-intensity exercise at least 2 days before the pre-testing, their nutrition was not controlled. Remer [53] showed a high impact of alkalizing-food intake on urine and blood pH values within 3 days, suggesting that nutrition should be standardized and controlled at least 3 days before anaerobic exercise testing in future investigations to reduce variability in urinary pH. In summary, we suggest that NaHCO_3_ ingestion during an altitude sojourn might be most effective when renal compensation of respiratory alkalosis is in progress or completed. However, to the best of our knowledge, the exact time course of renal compensation of hypoxia-induced respiratory alkalosis is unknown, and should be investigated in a controlled setting with at least 6 h of hypoxic exposure.

Notably, hypoxia-induced respiratory alkalosis is usually described as a desirable and important process that contributes to altitude adaption [54]. Chronic NaHCO_3_ ingestion thus contrasts with recommendations regarding the use of acetazolamide for the prevention of AMS when ascending to moderate and high altitudes [55]. Acetazolamide is a potent carbonic anhydrase inhibitor that increases minute ventilation and oxygenation and causes diuresis and renal [HCO_3_^−^] loss by enhancing central chemoreceptor output [56]. Via this mechanism, acetazolamide has been shown to provide prophylaxis for the symptoms of AMS in individuals ascending to high altitudes [55, 57]. Because NaHCO_3_ ingestion aims to compensate for the [HCO_3_^−^] loss rather than supporting hypoxia-induced diuresis and the associated renal [HCO_3_^−^] loss, we measured the AMS score every morning using the LLS in the present study, to control a potential higher risk of AMS due to NaHCO_3_ ingestion. However, chronic NaHCO_3_ ingestion had no significant effect on LLS values during the 7-day altitude sojourn, suggesting that it did not increase the risk of developing AMS symptoms. In addition, information on the effect of acetazolamide on exercise performance is still insufficient. Although acetazolamide was shown to impair submaximal and maximal exercise performances at sea level, its influences on submaximal and maximal exercise at altitude remain controversial [58]. We therefore conclude that NaHCO3 supplementation as an alkalotic buffer remains an interesting approach to alleviating acidic stress during exercise above the lactate threshold under hypoxic conditions, with no increase in the risk of AMS development.

The current dosage strategy may have been another possible reason for the lack of anaerobic performance enhancement by chronic NaHCO_3_ ingestion, which was contrary to our original hypothesis. We administered NaHCO_3_ according to a chronic schedule due to a lack of studies using chronic dosing schedules [16]; moreover, a chronic alkalotic state may meet the requirements of mountain sport disciplines better than single-dose NaHCO_3_, resulting in short-term performance enhancement. However, the effects of chronic NaHCO_3_ ingestion on anaerobic exercise performance improvements may have been hindered by the relatively long mountaineering exercise with altitude exposure, as well as the associated altitude adaption processes. Furthermore, most studies investigating the influence of NaHCO_3_ supplementation on anaerobic exercise performance under acute normobaric hypoxic conditions determined the individual time to peak [HCO_3_^−^] after NaHCO_3_ ingestion, and administered NaHCO_3_ in test trials at the participant’s pre-determined time to peak [HCO_3_^−^] to achieve the optimal performance changes [11, 16, 19]. The time course to peak blood [HCO_3_^−^] using liquid supplementation has previously been shown to range from 40 to 90 min [19]. Unfortunately, we were unable to assess the pre-determined time to peak [HCO_3_^−^] in the present study and we therefore decided to administer the daily NaHCO_3_ dose in 1 l of water 60 min before exercise testing. Although we assumed that this time frame met the requirements for maximizing the ergogenic effect of NaHCO_3_, it is possible that individualized supplementation strategies may be superior for the optimization of the performance-enhancing properties of NaHCO_3_.

It is also possible that the results might have differed if anaerobic performance tests had been performed at higher altitudes or for longer altitude exposures and associated daily NaHCO_3_ ingestion periods. We therefore intended to assess anaerobic performance after 7 days of NaHCO_3_ ingestion (HYP6) at 4554 m, but were unable to report the associated performance data due to computer crashes caused by high-altitude barometric pressure changes. Follow-ups to the current pilot study should thus focus on anaerobic performance tests at higher altitudes and after longer NaHCO_3_ ingestion periods, bearing in mind the potential computer-related problems caused by high altitude barometric pressure changes. Additionally, it has been proposed that performance of single sprints of short duration (up to 45 s) can be maintained in acute hypoxic conditions because of a shift toward anaerobic metabolism, whereas power output for tests with continuous or repeated high-intensity exercise longer than 45 s (such as the 3-min all-out critical power test and repeated sprints [11, 59, 60]), is often reduced in acute hypoxia [61, 62]. Therefore, we assumed that a 60-s continuous test protocol would be sufficient to assess changes in anaerobic exercise performance under hypoxic conditions. However, we presume that follow-up studies may involve the use of different test protocols, including assessments of all-out running for longer durations up to 3 min or repeated-sprint performance, to further investigate the results of impaired anaerobic exercise performance in hypoxia.

A further potential limitation of the present study and possible explanation for the lack of any significant effect of chronic NaHCO_3_ ingestion on ∆MF, ∆PF, ∆FI, and ∆La_max_ may be the low test-power of the comparisons between the bicarbonate and control groups. An a priori power calculation indicated that a sample size of three participants per group would allow the detection of differences between the groups, based on an earlier study that reported significantly improved force in a 60-s anaerobic performance cycling test under normoxic conditions after chronic NaHCO_3_ ingestion, with a statistical power of 57% [63]. However, we acknowledge that the use of data from the abovementioned investigation resulted in a surprisingly low calculated sample size for detection of possible changes. Indeed, smaller effect sizes were found within the current investigation and a sample size of 10 participants resulted in a test power of 10% (6–14%) for the effect of NaHCO3 ingestion on PTSR-related parameters. In contrast, power calculations for the metabolic parameters measured in this study were associated with higher power values (11–99.8%). This indicates sufficient test power to analyze the effect of NaHCO_3_ supplementation, but an underpowered trial in terms of determining the effect of anaerobic exercise at altitude, making the detection of significant difference in ∆MF, ∆PF, ∆FI, and ∆La_max_ between the bicarbonate and control groups highly unlikely. These small effects meant that we would probably not be able to detect differences in these parameters with the current sample size of 10 participants, and could therefore not exclude a type 2 error within our interpretation.

Furthermore, the high individual variations represented by individual trajectories in Fig. 2 in response to anaerobic exercise at altitude may have contributed to the lack of power for the effect of anaerobic exercise at altitude. Individual variations in this context may represent a previously suggested responder vs. non-responder phenomenon to intervention with NaHCO_3_ supplementation [64, 65] or exercise performance changes under hypoxic conditions [66, 67]. Another explanation for variations in the response to anaerobic exercise at altitude may be an unfamiliar exercise pattern, such that the participants were unable to properly implement the test instructions. It has already been shown that tethered sprinting reduces maximal velocity, flight time, and stride length, and increases contact time, compared with free sprinting [68, 69]. Therefore, it is possible that the PTSR test pattern may have prevented our participants from sprinting to their full potential, meaning that the maximal performance measurement may not reflect the participants’ true maxima. In addition, the inclusion of a single female participant in each group may have contributed to variability in the effect of anaerobic exercise at altitude; a general sex-related difference may have introduced additional heterogeneity. We therefore also performed statistical analyses on PTSR-related performance parameters in male participants alone, but found no significant differences in the outcomes from the results of analyzing the complete groups. Therefore, we decided to report the data from both groups, including the female participants, to achieve higher study power.

Nevertheless, we assumed that the possible undetected differences in PTSR-related performance parameters were likely to be too small to contribute to an anaerobic performance enhancement, and influences of other factors may have negatively affected the ergogenic effects of NaHCO_3_ ingestion. This assumption was supported by the higher [HCO_3_^−^] and BE values pre- and post-PTSR in the bicarbonate group compared with the control group, but the lack of any significant difference in exercise-induced difference between pre- and post-PTSR values. Recent studies reported an increased glycolytic energy contribution to exercise and improved anaerobic exercise performance following NaHCO_3_ ingestion under normoxic [70] and acute hypoxic conditions [16]. It has also been suggested that changes in blood pH and [HCO_3_^−^] are greater during exercise with NaHCO_3_ ingestion and the associated elevation of pre-exercise [HCO_3_^−^] and BE values, which is supposed to explain the increased anaerobic exercise performance in acute hypoxic conditions following NaHCO_3_ ingestion [19]. However, our data do not support these assumptions because despite higher [HCO_3_^−^] and BE values pre- and post-PTSR following NaHCO_3_ ingestion, only the exercise-induced difference between pre- and post-PTSR values for ∆pH_b_ differed between conditions, indicating similar glycolytic energy contributions and exercise performance outputs irrespective of NaHCO_3_ ingestion, but a possible difference in respiratory contributions resulting in less-pronounced acidosis following NaHCO_3_ ingestion.

Participant acclimatization may also have had a negative influence of the ergogenic effect of NaHCO_3_ ingestion by negating the additional acidic load apparent in unacclimatized individuals. Although the results for s_a_O_2,_ [HCO_3_^−^], and BE do not suggest that our participants were already fully acclimatized at HYP3 when they performed the PTSR test at altitude, further studies are needed to prove the assumptions raised in the present pilot study. Furthermore, the unexpected lack of an ergogenic effect of NaHCO_3_ could be explained by the theory of strong ion difference (SID) [71]. The present findings refer to the Henderson-Hasselbach approach, which assumes that blood pH is determined by changes in [H^+^] and [HCO_3_^−^]. In contrast, the SID approach refers to the intra- and extracellular ions (e.g. chloride, potassium, sodium) and describes the difference between the concentrations of strong cations and strong anions. The SID is also suggested to have an independent effect on blood pH, and thus impair muscle performance by altering intra- or extra-cellular pH [71]. The SID approach may therefore explain the exercise-induced difference between pre- and post-PTSR values for ∆pH_b_ between the bicarbonate and control groups with simultaneously similar developments of changes in PTSR-related parameters, [HCO_3_^−^], and BE. However, this conclusion should be interpreted with caution because we did not calculate the SID values in the present study, and future studies are needed to examine the influence of changes in the SID on anaerobic exercise performance at altitude.

PTSR performance parameters in this study might also have been influenced by gastro-intestinal (GI) disturbances. Negative GI symptoms caused by bicarbonate ingestion have been reported in the literature [21, 72, 73] and GI discomfort is suggested to have ergolytic effects on anaerobic performance [15, 19]. Unfortunately, we did not carry out any structured monitoring of GI discomfort in the current participants, which represents a limitation of this study. However, we asked the subjects—in daily individual unstructured interviews—about any GI disturbances after consuming the NaHCO_3_ solution; most participants in the bicarbonate group reported GI complaints after NaHCO_3_ ingestion. We mainly attributed these GI disturbances to the dose of NaHCO_3_ (0.3 g/kg). Although this dose has been recommended for NaHCO_3_ supplementation under normoxic and hypoxic conditions [15, 16, 39–42], smaller doses have been suggested for participants who display severe GI symptoms after NaHCO_3_ ingestion [16]. Given that GI discomfort seems to increase with increasing NaHCO_3_ dose [72], we decided to reduce the dose to 0.15 g/kg body mass on day five of supplementation, after which the subjects’ reported GI symptoms decreased. However, in retrospect, we would potentially recommend a reduction to the common dose of 0.20 g/kg body mass, rather than the pronounced reduction (by 50%) to 0.15 g/kg body mass. Finally, within the present study, the PTSR test under hypoxic conditions was performed at a dose of 0.3 g/kg, and the performance outputs may have been inhibited due to GI discomfort. In addition, it must be noted that GI problems are common at high altitude and are often reported, regardless of NaHCO_3_ ingestion [74, 75]. The reported GI discomfort in the present study may thus have been due to both NaHCO_3_ ingestion and altitude exposure. Further investigations under hypobaric hypoxic conditions should thus be performed using NaHCO_3_ at a lower dose or in a different dosage form; these should include controls for and monitoring of GI upset using structured daily self-reports [16]. Different dosage forms and strategies that have been reported to reduce GI side effects include the use of tablets or capsules (instead of liquid supplementation), serial loading [76], and co-ingestion of NaHCO_3_ with water and a high-carbohydrate meal [14]. Future studies should include the use of a placebo supplement for the control group; this aspect was not implemented in the present study and therefore constitutes a limitation of the study.

Finally, another limitation of the study is that it was not double-blinded. Although the study was originally designed with this in mind, the serious gastrointestinal problems reported by the participants forced us to inform the study investigators and participants regarding group affiliations, prior to reducing the NaHCO_3_ dose. Therefore, the present study provides the first results in this field, but further research is needed to confirm the findings of this investigation with regard to the effects of chronic NaHCO_3_ ingestion on anaerobic exercise performance under hypobaric, hypoxic conditions.

### Practical applications

Although mountaineering is mainly associated with aerobic performance [29], the results of the current study will be applicable to other mountain sports disciplines performed at moderate to high altitudes. Previous studies demonstrated the need for a high level of anaerobic power during steep climbs and sprints in cross-country ski races [30], and cross-country sprint disciplines with maximal-effort durations of 2.5–3 min are also expected to have a significant anaerobic contribution [31]. Moreover, alpine ski races last for 45 s to 2 min, and anaerobic fitness has been identified as being of primary importance in alpine skiing [32, 33]. To the best of our knowledge, no studies have examined ski mountaineering, though the findings for alpine skiing may be transferable to ski touring and ski mountaineering. Additionally, anaerobic power has been suggested to be an important determinant of performance in cross-country and downhill mountain biking and transalpine challenges [34, 35, 77]. Unfortunately, no studies have examined the physiological requirements of disciplines such as multi-pitch rock, mixed, or ice climbing, and recent studies have focused on the physiology of difficult rock or indoor climbing. Performance in single-pitch climbing disciplines is mainly determined by anaerobic power and muscular strength [36, 37]; it might be necessary to consider whether multi-pitch climbing requires greater anaerobic power due to longer exercise duration. However, further studies are required to support these assumptions. The above-mentioned sport disciplines are thus affected by hypoxia-induced reduced exercise performance at altitude and may therefore benefit from a dietary strategy involving NaHCO_3_ ingestion to improve anaerobic exercise performance.

## Conclusion

Due to methodological uncertainties, the present randomized controlled trial should be considered as a pilot presenting first results examining the effects of chronic NaHCO_3_ supplementation on anaerobic exercise performance during an altitude sojourn. The principal finding was that oral chronic NaHCO_3_ ingestion did not affect hypoxia-induced performance changes (∆MF, ∆PF, ∆FI) or ∆La_max_ changes, but significantly increased the early-morning pH_b_, [HCO_3_^−^], and BE values, which partially represent the blood buffering capacity. A higher alkalotic state of the acid-base balance prior to exercise under hypoxic conditions is often associated with higher performance outputs and higher maximum blood lactate values after high-intensity exercise. Explanations for the apparent lack of any ergogenic effect of NaHCO_3_ ingestion include pre-acclimatization, the time course of hypoxia-induced renal [HCO_3_^−^] compensation, changes in intra- and extracellular ions others than [H^+^] and [HCO_3_^−^], or GI disturbances caused by NaHCO_3_ ingestion. Further, the present study provides important practical advices for future field investigations in this research area, such as a reduction of commonly used sodium bicarbonate doses to prevent for exercise decrements due to gastro-intestinal symptoms and the computer crashes caused by high-altitude barometric pressure changes.

## Data Availability

The dataset supporting the conclusions of this article is available in the figshare data repository [doi: 10.6084/m9.figshare.8937995].

## Abbreviations

AMS: Acute mountain sickness
BE: Base excess
BIC: Bicarbonate supplementation group
CON: Control group
DIFF PTSR: Difference between pre- and post-PTSR values
FI: Fatigue index
GI: Gastro-intestinal
[H^+^]: Hydrogen cation
[HCO_3_^−^]: Blood bicarbonate
HYP: Hypoxic conditions
La_max_: Maximum post-exercise lactate concentration
LLS: Lake Louise score
MF: Mean force
NaHCO_3_: Sodium bicarbonate
NOR: Normoxic conditions
P_I_O_2_: Oxygen pressure in the inspired air
PF: Peak force
pH_b_: Blood pH
pH_u_: Urinary pH
PCO_2_: Carbon dioxide partial pressures
PO_2_: Oxygen partial pressures
PTSR: Portable tethered sprint running test
SID: Strong ion difference
